# Quantifying Bottoming Out in Inferior Pedicle Breast Reduction: A Longitudinal ThreeDimensional Study

**DOI:** 10.1093/asjof/ojag138.017

**Published:** 2026-07-24

**Authors:** Hayeem Rudy, Evan Rothchild, Caroline Baker, Teresa Benacquista

## Abstract

**Background:**

“Bottoming Out” is a recognized phenomenon in inferior pedicle breast reduction wherein gravitational and healing forces increase the volume of the lower pole of the breast while reducing the volume of the superior pole of the breast. To date, there have been no studies that have attempted to quantify these changes from a temporal, geometric, and volumetric standpoint. This problem is well suited for study with threedimensional imaging, particularly with the 3D scanner of the iPhone. Our group has previously validated the iPhone against the Canfield Vectra M1 as an accurate system for capturing 3D data of the breast. The purpose of this study was to quantify volumetric and geometric changes in breast shape over time in patients undergoing inferior pedicle breast reduction surgery.

**Methods:**

We conducted an IRB-approved prospective study that included patients undergoing inferior pedicle BRM performed by a single surgeon from June 2023 to September 2024. Patients were enrolled and scanned at 1 week, 1 month, and 3 month, and 6 month postoperative visits using the iPhone 3D scanner. All 3D images obtained were exported and postprocessed using Blender (Blender Foundation, Vienna, Austria) opensource software, and imported into the Vectra Analysis Module software (Canfield Scientific). Anatomical breast landmarks were manually established and utilized to calculate each breast's total, upper pole, and lower pole surface area. Analysis of surface area changes over time was conducted.

**Results:**

A total of 15 patients were included through 6 months postoperatively. At 1 month, compared to the baseline postoperative visit, the inferior pole surface area increased by an average of 1.78%, while the superior pole surface area increased by 4.03%. At 3 months, the inferior pole surface area increased by 6.17% compared to baseline, while the superior pole surface area decreased by 6.72% compared to 1 week. At 6 months, the inferior surface pole surface area increased by 7.02% on average, compared with a decrease in the surface areas of the lower pole by 8.11%. At six months, the nipple to IMF distance increased by approximately 1.3cm ± 0.32 and the sternal notch to nipple distance increased by 1.0cm ± 0.2.

**Discussion:**

Our findings indicate that at the six-month postoperative timepoint there is a 7% percent increase in the surface area of the lower pole and a 8% reduction in the surface area of the upper pole, on avergae. Similarly, key landmarks including the vertical limb (NIMF) lengthened by approximately 1.3cm and the sternal notch to nipple distance increases by 1cm. These findings identify that the "bottoming out" phenomenon becomes apparent between 1 month and 3 month postoperative mark and that upper pole fullness decreased on average by 6 percent when compared to the on-table result. We will continue to follow patients enrolled in this study at their 1 and 2 year follow up periods.

